# Poisonous Effects of Carbolic Acid

**Published:** 1870-09

**Authors:** 


### Poisonous Effects of Carbolic Acid.

The *Edinburgh Medical Journal* says: Professor Bardeleben found that when externally applied in surgical cases, carbolic acid was absorbed, and acted poisonously in about one case out of ten. This poisonous action was revealed often so early as the second day, by a peculiar effect on the urine, which, pale at first, became gradually darker on standing. No albumen was present in the urine, but the patients lost appetite and strength. He recommends as a substitute the sulphocarbolate of xinc, first employed by Wood. Mr. Lister states that he has never observed the peculiar dark urine since the paste was replaced by the lac plaster.

Dr. J. Wallace applied carbolic oil (1 to 8) to an abcess connected with morbus coxæ, in a child aged five. In about two months' time it was remarked that vomiting and dysphagia invariably followed each dressing, and on examining the urine, he found it to possess a dark smoky tint, very similar to the appearance of the urine in bad scarlatinal nephritis. Nitric acid added to the boiling urine threw down a heavy dark precipitate. No trace of albumen. This deposit of pigment invariably appeared after each dressing with the carbolic acid, and disappeared again in a few days. A fortnight after the above symptoms were noted, he adopted Prof. Lister's most recent method of carbolic dressing by oilskin, coated with dextrine and shell lac, and carbolic acid plaster; matters became more favorable, and the urine resumed its normal appearance. (*British Medical Journal,* April 30th). Dr. Lightfoot, in the same

Journal, reports a case in which alarming symptoms, resembling those of pyæmic poisoning, clearly resulted from the application of a weak aqueous carbolic lotion (1 to 50). The symptoms were developed three successive times when the lotion was employed, and gradually subsided on its removal. Vomiting was dangerously severe, so that the patient's life was almost despaired of, but the urine was not darkened in color. Numerous observers have recently met with cases of poisoning in connection with the use of carbolic acid, and it is very necessary to observe caution as to the too free external use of this agent. The *black* or darkened urine, which is the most constant symptom, has been shown to occur in an equally marked form, whether tar, or some colorless preparation of it be the agent employed. The exact cause of the coloration is still an open question, but it is at least probable that the coloring matter is not derived from the blood, The constitutional disturbance is sometimes very grave, and seems to bear some connection with different forms of solution of carbolic acid, the lac plaster appearing to be the safest, while a weak watery solution, freely used, apparently involves the most risk.—*Medical and Surgical Reporter.*

